# Antioxidant Activity and Inhibitory Effect on Nitric Oxide Production of Hydroponic Ginseng Fermented with *Lactococcus lactis* KC24

**DOI:** 10.3390/antiox10101614

**Published:** 2021-10-13

**Authors:** Yerim Chung, Ji-Young Park, Ji-Eun Lee, Kee-Tae Kim, Hyun-Dong Paik

**Affiliations:** Department of Food Science and Biotechnology of Animal Resources, Konkuk University, Seoul 05029, Korea; yerimchung@daum.net (Y.C.); parkgos2@naver.com (J.-Y.P.); ljesym@naver.com (J.-E.L.); richard44@hanmail.net (K.-T.K.)

**Keywords:** hydroponic ginseng, fermentation, probiotics, antioxidant activity, nitric oxide

## Abstract

*Panax ginseng* Meyer is used as a medicinal plant. The aim of this study was to ferment hydroponic ginseng with *Lactococcus lactis* KC24 and confirm its antioxidant activity and inhibitory effect on nitric oxide (NO) production. Flavonoid and phenol contents in fermented ginseng extracts were measured. Antioxidant activity was measured by DPPH, ABTS, reducing power, FRAP and β-carotene assays. Additionally, inhibitory effects on NO production and toxicity of the fermented extract were determined using RAW 264.7 cells. Phenol and flavonoid contents increased as the fermentation time increased, and the contents were higher in hydroponic ginseng than in soil-cultivated ginseng. The DPPH assay revealed that the antioxidant activity of the 24 h fermented extract significantly increased from 32.57% to 41% (*p* < 0.05). The increase in antioxidant activity may be affected by an increase in phenol and flavonoid contents. At 1 mg/mL solid content, the 24 h fermented hydroponic ginseng extract inhibited NO production from 9.87 ± 0.06 μM to 1.62 ± 0.26 μM. In conclusion, the increase in antioxidant activity affects the inhibition of NO production, suggesting that fermented hydroponic ginseng may be used in the industries of functional food and pharmaceutical industry as a functional material with anti-inflammatory effects.

## 1. Introduction

*Panax ginseng* Meyer is widely known as a traditional pharmaceutical plant in Asia, especially in Korea and China. Therefore, diverse studies have already reported the bio-functionality of ginseng, such as its neuroprotective effects [1], protection against myocardial injury [2], inhibition of nitric oxide (NO) production [3], antibacterial effects and antioxidant activities [4]. However, ginseng grown in the soil has a long cultivation period and is, therefore, not economical [5]. In addition, because of the long cultivation period, the quality of ginseng is greatly affected by the cultivation environment [6]. Typically, crops are easily damaged by pests, and pesticide residue problems have been identified. On the other hand, hydroponically cultivated ginseng is more economical as it is generally cultivated for only 1 to 2 years, which is a shorter cultivation period than that of soil cultivation [6,7]. Additionally, as it is generally grown in a greenhouse, the cultivation environment can be controlled arbitrarily [8], and ginseng is not affected by insects [9], allowing its growth without the use of chemical pesticides [10].

Ginseng leaves are known to have better antioxidant properties than ginseng roots [11,12,13]. Accordingly, all parts of hydroponic ginseng, including its leaves and roots, were used as a sample in this study. Interestingly, the size of hydroponic ginseng is smaller than that of soil-cultivated ginseng. Nevertheless, hydroponic ginseng can be used as a good medicinal material. Our previous study demonstrated that hydroponic ginseng and its fermented products have various health benefits, including antioxidant activity, anti-adipogenic and anti-inflammatory properties [14]. 

Fermentation is a process that has attracted attention of food and pharmaceutical industries worldwide. Fermentation has provided important and diverse benefits from an early historical period and has not only made it possible for humans to manage food as a resource, but also improved the functional properties of agriculture. Indeed, fermentation lengthens the preservation period of food, thereby enhancing its safety and stability [15]. In addition, fermentation lowers the toxicity of highly toxic foods or improves the flavor to increase economic efficiency. This characteristic of fermentation has drawn interest in the use of fermentation in almost every culture worldwide. In the past, the fermentation process was mainly found in the traditional foods of each country. Recently, however, through probiotic fermentation, foods with better health benefits than conventional foods are being developed. Probiotics are related to food fermentation and are generally recognized as safe. In addition to enhancing nutritional composition, taste, flavor and shelf life, fermented foods with probiotics promote the health status of individuals through prophylactic and therapeutic effects [16]. Probiotic fermentation has been studied for general dairy products, processed meat products [17], beverages [18] and plant foods [19]. 

In Table 1, *Lactococcus lactis* KC24 that used in our study, showed excellent probiotic properties, including artificial bile salt (85% survival ratio) and intestinal adhesion (18% in xylene, 54% in chloroform and 4% in ethyl acetate) [20]. Additionally, this strain had proven to have many bio-functional properties in previous study. For instance, it increased the antioxidant ability of the cells (80%), as indicated by the DPPH assay, and increased cytokines ex vivo in immunity evaluation. Furthermore, the strain is safe for human health as *L. lactis* KC24 does not produce β-glucuronidase, which generates toxic aglycones and carcinogens from carbohydrates [20]. *L. lactis* KC24 even showed higher neuroprotective effect than *Lactobacillus rhamnosus* GG, the commercial probiotics [21].

Oxidative stress occurs as reactive oxygen species (ROS) levels exceed the intrinsic antioxidant capacity of cells. At moderate levels, ROS can function as signal transmitters, but, at excessive levels, ROS can be detrimental to cells because of their high reactivity. Oxidative stress refers to a condition in which there is a serious imbalance between ROS generation and the antioxidant defense system.[22] ROS mediates cellular signaling processes in inflammatory responses, hypertension and hyperlipidemia, directly induces tissue damage and reduce vascular function. Moreover, the signaling action of nitric oxide (NO) is always dependent on the concentration of ROS in the body that can remove NO and the activity of metalloproteins. Oxidation reactions accompanied by NO depletion not only affect NO signaling but also generate secondary oxidation, nitration and nitrosation/nitrosylation substances, which exhibit unique inflammatory cell signaling properties [23]. 

The fermentation of ginseng, a traditional Asian medicinal herb, has recently attracted attention. Fermentation of ginseng with probiotics has been reported to increase its antidiabetic effects [24], antioxidant activity [25] and NO scavenging activity [26]. Research on the fermentation of hydroponic ginseng has been on the rise since 2015. In a previous study, the antioxidant activity and anti-inflammatory effects of fermented hydroponic ginseng was demonstrated [27,28]. Consequently, fermenting hydroponically cultivated ginseng with probiotics, which is known to have better functionality than soil-cultivated ginseng, is considered promising. Therefore, the aim of this study was to determine the difference of the antioxidant activity and inhibitory effects on NO production of hydroponic ginseng extracts and those of the fermented extracts to improve their bio-functionalities.

## 2. Materials and Methods

### 2.1. Microorganisms and Materials

2-year-old hydroponic ginseng (HG) and 6-year-old soil-cultivated ginseng (SG) were purchased from Ginseng Well-Life Cooperative (Gwangju, Korea) and Sangdo-Insam Company (Yeongju, Korea), respectively. 

*Lactococcus lactis* KC24 was isolated from kimchi and stored at −70 °C before use. The strain was incubated in De Man, Rogosa and Sharpe (MRS, BD Bioscience, Franklin Lakes, NJ, USA) broth at 37 °C for 15 h before inoculation into ginseng extracts for fermentation.

RAW 264.7 cells (murine macrophage cells; KCLB 40071) were purchased from the Korean Cell Line Bank (Seoul, Korea). Dulbecco’s modified Eagle’s medium (DMEM; Hyclone TM, Logan, UT, USA) containing heat-inactivated 10% fetal bovine serum (FBS; Hyclone TM, Logan, UT, USA) and 1% penicillin-streptomycin (Hyclone TM, Logan, UT, USA) is used as its medium and incubated at 37 °C incubator with 5% CO_2_ (MCO-18AIC, SANYO, Osaka, Japan).

### 2.2. Chemicals

MRS broth and agar media were purchased from Difco Laboratories (Detroit, MI, USA). The reagent 3-[4,5-dimethylthiazol-2-yl]-2,5 diphenyl tetrazolium (MTT) was obtained from Sigma-Aldrich Co. (St. Louis, MO, USA). DMEM, penicillin-streptomycin solution and FBS were purchased from Hyclone TM. 

### 2.3. Fermentation of HG and SG Extracts

HG and SG were extracted twice with 50% ethanol using OCOO, double boiling pot and concentrated using a vacuum evaporator (EYELA N-1000V, Tokyo, Japan). All parts of HG and SG were used in this study. For fermentation, extracts at a concentration of 10 mg/mL were prepared in distilled water (DW) and adjusted to pH 6.5 to drive optimal growth [29]. The samples were autoclaved at 90 °C for 30 min, and 1% *L. lactis* KC24 was inoculated in ginseng extracts for fermentation. Ginseng extracts were fermented in 37 °C aerobic condition without stirring for 12 and 24 h. The initial concentration of *L. lactis* KC24 was set to ± 6.5 log CFU/mL. The non-fermented and fermented ginseng extracts were centrifuged and filtered with a 0.45 µm membrane filter. Filtered samples were used for subsequent experiments.

### 2.4. Measurement of Total Flavonoid Content

Total flavonoid content (TFC) was measured by an aluminum chloride assay [30]. The concentration of fermented and non-fermented ginseng extracts was adjusted as 2.5 and 5 mg/mL using DW. Mixtures containing 100 μL of a ginseng extract, 20 μL of 5% (*w*/*v*) NaNO_2_ and 800 μL of 60% (*v*/*v*) ethyl alcohol were prepared and incubated for 6 min. Then, 20 μL of 10% (*w*/*v*) AlCl_3_ and 60 μL of 4% (*w*/*v*) NaOH were added to the mixtures. After 30 min incubation at 25 °C, absorbance of the ginseng extracts was measured at 405 nm. Quercetin was used as a standard for the construction of the calibration curve, and TFC was calculated as quercetin equivalents (mg)/dry weight (g). The equation of the quercetin calibration curve was as follows:

$$Y=1.298X-0.0644,{\;R}^{2}=0.9975$$

### 2.5. Measurement of Total Phenol Content

Total phenol content (TPC) was analyzed using the Folin-Ciocalteu method [29] with minor modifications. The concentration of fermented and non-fermented ginseng extracts was adjusted as 2.5 and 5 mg/mL using DW. Mixtures containing 50 μL of a ginseng extract and 1 mL of 2% (*w*/*v*) Na_2_CO_3_ were vortexed and then allowed to sit for 3 min. A 50% (*v*/*v*) Folin–Ciocalteu solution (50 μL) was added and mixed. The mixtures were incubated for 30 min at 25 °C, and absorbance was measured at 750 nm. Gallic acid was used as a standard for the construction of the calibration curve, and TPC was calculated as gallic acid equivalents (mg)/dry weight (g). The equation of the gallic acid calibration curve was as follows:

$$Y=1.2715X-0.0257,\;R^{2}=0.9966$$

### 2.6. Measurement of Antioxidant Activity

#### 2.6.1. Diphenyl-1-picryl-hydrazyl Radical Scavenging Activity

The 2,2-diphenyl-1-picryl-hydrazyl (DPPH) radical scavenging activity in both non-fermented and fermented ginseng extracts was determined as described by Yang et al. [30], with minor modifications. A 0.4 mM DPPH solution (750 μL) solubilized in ethanol was added to 150 μL of non-fermented and fermented ginseng samples. The mixtures were vortexed and incubated at 25 °C for 30 min in the dark. The absorbance of the mixture was measured at 517 nm, and the DPPH radical scavenging activity was calculated as follows:

$$\mathrm{DPPH}\;\mathrm{radical}\;\mathrm{scavenging}\;\mathrm{activity}\;\left(\%\right)=\left(1-\frac{A_{s}}{A_{c}}\right)\times 100.$$

where A_s_ and A_c_ are the absorbance values of samples and control, respectively.

#### 2.6.2. Azinobis-(3-ethylbenzothiazoline-6-sulfonic acid) Radical Scavenging Activity

The 2,2′-azinobis-(3-ethylbenzothiazoline-6-sulfonic acid) (ABTS) radical scavenging activity of the ginseng extracts was evaluated by a modified method described by Kim et al. [31]. The ABTS solution was prepared by mixing 7 mM potassium persulfate and 14 mM ABTS, diluting in 0.1 M potassium phosphate buffer (pH 7.4) and incubating at 20–25 °C in the dark for 12–16 h. Reacted ABTS solution was diluted to a final absorbance of 0.7 ± 0.01 at 734 nm in 0.1 M potassium phosphate buffer (pH 7.4). Next, mixtures containing 50 μL of a ginseng extract and 950 μL of ABTS solution were incubated for 15 min at room temperature. The absorbance of the non-fermented and fermented ginseng extracts was measured at 734 nm, and the ABTS radical scavenging activity was calculated as follows:

$$\mathrm{ABTS}\;\mathrm{radical}\;\mathrm{scavenging}\;\mathrm{activity}\;\left(\%\right)=\left(1\text{–}\frac{A_{s}}{A_{c}}\right)\;\times 100.$$

where A_s_ and A_c_ are the absorbance values of samples and control, respectively.

#### 2.6.3. Reducing Power Assay

The reducing power assay was conducted as reported method [32] with modification. Briefly, 50 µL of a ginseng extract was mixed with 250 µL of 0.2 M sodium phosphate buffer (pH 6.6) and 250 µL of 1% potassium ferricyanide. The mixtures were incubated at 50 °C water bath for 20 min, added with 250 µL of 10% trichloroacetic acid, and then centrifuged at 6000 rpm for 5 min. The upper layer of the supernatant (500 µL) was mixed with 100 µL of 0.1% ferric chloride and 400 µL of DW. The mixtures were then allowed to react for 30 min at 20–25 °C in the dark. Absorbance was measured at 700 nm using a spectrophotometer (Optizen 2120UV Plus; Mecasys Co., Ltd, Daejeon, Korea). L-Cysteine was used as the standard. The equation of the L-Cysteine standard curve was as follows:

$$Y=0.017X+0.143,{\;R}^{2}=0.9913$$

#### 2.6.4. Ferric Reducing Antioxidant Power Assay

Antioxidant activity of the ginseng extracts was determined according to a previously reported method [33] with modifications. A solution was prepared using 300 mM acetate buffer (pH 3.6), 10 mM 2,4,6-tri[2-pyridyl]-s-triazine solution and 20 mM ferric chloride solution in a 10:1:1 ratio and pre-heated in a 37 °C water bath for 15 min. Ginseng extracts (50 µL) were mixed with 950 μL of the solution and incubated at 20–25 °C for 30 min in the dark. Absorbance was measured at 593 nm using a spectrophotometer (Optizen 2120UV Plus; Mecasys Co., Ltd, Daejeon, Korea). A ferrous sulfate (FeSO_4_) solution was used as the standard. The equation of the FeSO_4_ standard curve was as follows:

$$Y=0.054X+0.3279,{\;R}^{2}=0.9983$$

#### 2.6.5. β-Carotene Bleaching Assay

The β-carotene bleaching assay was performed as described by Kassim et al. [34] with some modifications. A β-carotene solution was prepared with 44 μL of linoleic acid, 2 mg of β-carotene and 200 μL of Tween 80 in 10 mL of chloroform in a round-bottom flask. The chloroform in the solution was evaporated at 40 °C using a rotary vacuum evaporator (EYELA N-1000V, Tokyo, Japan). Then, 100–150 mL of DW was added to the evaporated solution and diluted until the absorbance of the solution was 1.8 at 470 nm. Each ginseng extract sample (100 μL) was mixed with 400 μL of β-carotene solution and incubated in a 50 °C water bath for 2, 4 and 6 h. The absorbance of the samples was determined at 470 nm. The antioxidant activity was calculated using the following equation:

$$\beta -\mathrm{Carotene}\;\mathrm{bleaching}\;\mathrm{inhibitory}\;\mathrm{activity}\;(\%)=\frac{A_{\mathrm{sample},2h}\;–{\;A}_{\mathrm{control},2h}}{A_{\mathrm{control},0h}\;{–\;A}_{\mathrm{control},2h}}\;\times 100.$$

where A_sample,2h_ and A_control,2h_ are the absorbance values of samples and controls after the 2 h incubation, respectively. As the samples were also incubated for 4 h and 6 h, the values of A_sample,2h_ and A_control,2h_ were replaced with A_sample,4h_, A_control,4h_ or A_sample,6h_, A_control,6h_ depending on the incubation time. A_control,0h_ is the absorbance of DW at the initial incubation time.

### 2.7. Nitric Oxide (NO) Production and Cell Viability Assay Using MTT Assay

RAW 264.7 cells were cultured in DMEM (Hyclone TM, Logan, UT, USA) with 10% (*v*/*v*) FBS and 1% (*v*/*v*) streptomycin/penicillin solution (Hyclone TM, Logan, UT, USA). The amount of NO in the cell culture medium was evaluated as previously described [25]. 100 µL of RAW 264.7 cells (2 × 10^6^ cells/mL) were plated into 96-well plates, incubated for 2 h and added with 50 µL of ginseng extract or 50 µL of lipopolysaccharide (LPS, 4 µg/mL; positive control). After a 24 h incubation, 100 µL of the supernatant of the cell culture medium was added to new 96-well plates with 100 µL of Griess reagent, and the absorbance was measured at 540 nm using a microplate reader (Molecular Devices, Sunnyvale, CA, USA). NO production was determined by comparing with sodium nitrate as the standard. 

The MTT assay [30] was performed to determine the toxicity of HG extracts on RAW 264.7 cells. After measuring NO concentration in the medium, 100 μL of the 0.5 mg/mL MTT (Sigma-Aldrich Co., St. Louis, MO, USA ) solution diluted with DMEM was added to each well and incubated for 1 h. Supernatants were removed, and 100 μL dimethyl sulfoxide was added to each well for 15 min to dissolve the formazan crystals. Absorbance was measured at 570 nm using a microplate reader (Molecular Devices, Sunnyvale, CA, USA). Cell viability was calculated and compared with the results of the control group, which was not treated with extracts, using the following equation:

$$\mathrm{Cell}\;\mathrm{viability}\;(\%)=\frac{A_{s}}{A_{c}}\times 100$$

where A_s_ and A_c_ are the absorbance values of samples and controls, respectively.

### 2.8. Statistical Analysis

All experiments were conducted at least three times on triplicate samples. The values were expressed as mean ± standard deviation, and one-way analysis of variance was performed using the SPSS software (version 20, SPSS Inc., Chicago, IL, USA). Duncan’s multiple range test was performed to test for significant differences between treatment groups at *p* < 0.05.

## 3. Results

### 3.1. Changes in the Number of Bacteria and pH during Fermentation

**Figure 1:**
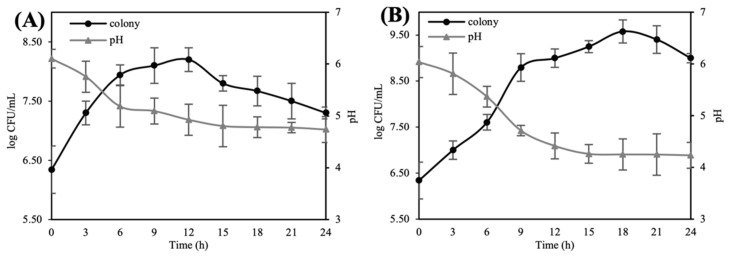
Figure **1.** Growth curves and change in pH of the ginseng extracts during fermentation. (**A**) 2-year-old hydroponic ginseng extract and (**B**) 6-year-old soil-cultivated ginseng extract. ●, viable cell count; ▲, pH. Each viable cell count and pH of ginseng extracts was measured every 3h from the beginning of fermentation until 24h fermentation. The bars represent mean ± standard deviation of at least triple repetitive analysis.

After HG and SG were extracted using 50% ethanol and concentrated, the ginseng extracts were adjusted to pH 6.5 to induce an optimum growth rate. During fermentation with 1% *L. lactis* KC24, each ginseng extract was sampled every 3 h to measure colony counts and pH changes (Figure 1). Both HG and SG extracts showed a similar trend; however, SG showed more colonies in its stationary phase. Owing to more lactic acid bacteria, the fermented SG extract had a lower pH than the fermented HG extract. 

### 3.2. Total Phenol and Flavonoid Contents

As shown in Table 2, TPC and TFC in HG and SG extracts increased during fermentation. TPC in 12 h and 24 h fermented HG extracts increased by 6.1% and 4.1%, respectively, and were higher than those of the non-fermented extracts. Similarly, total content of flavonoids, a class of polyphenolic compounds, in ginseng extracts was also measured and found results similar to those of TPC. TFC of non-fermented HG increased by 3.1% after 12 h of fermentation but decreased by 2.9% after 24 h of fermentation. TPC and TFC in SG extracts increased with increasing fermentation time. This pattern was not observed for fermented HG.

### 3.3. Antioxidant Activity of Fermented HG and SG Extracts

**Figure 2:**
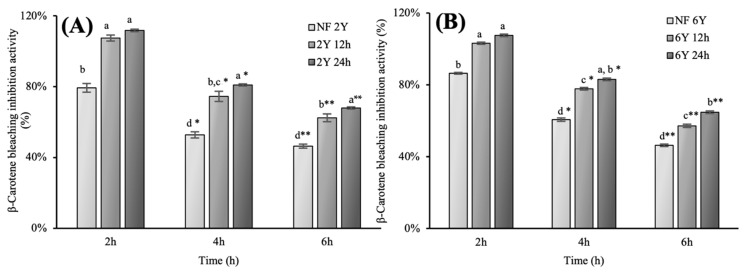
Inhibition of lipid-oxidation activities of ginseng extracts measured by β-carotene bleaching assay. (**A**) 2-year-old hydroponic ginseng (5 mg/mL) during fermentation; (**B**) 6-year-old soil-cultivated ginseng (5 mg/mL) during fermentation.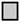, non-fermented;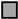, 12 h fermented;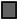, 24 h fermented. The graphs are presented with triplicate experiments as mean value ± S.D. Different letters (^a,b^, 2 h; ^a^*^–d^*, 4 h; ^a^**^–d^**, 6 h.) on the error bars represent significant differences between same treated time (*p* < 0.05).

Antioxidant assays were conducted using different concentrations of ginseng extracts (2 and 5 mg/mL). As shown in Table 3 and Table 4, the DPPH radical scavenging activity of 24h fermented HG extracts was significantly higher than that of non-fermented extracts. However, 12 h fermented SG extracts showed decrease in DPPH radical scavenging activity. Contrarily, ABTS radical scavenging activity of all ginseng extracts was high (above 80%). In particular, 24 h fermented HG and SG extracts exhibited highest antioxidant capacity (88.82%). In contrast with the DPPH assay, each extract only showed an increase in antioxidant activity after fermentation. Reducing power and ferric reducing antioxidant power assay, the trend of result was similar to other previous antioxidant activities, though the rise after fermentation was smaller than other assays. For β-carotene bleaching inhibition assay, fermented hydroponic ginseng extract showed higher antioxidant activities compared to that of non-fermented HG extract, fermented SG extract and non-fermented SG extract (Figure 2). In general, antioxidant activities of fermented HG and SG extracts were greater than those of non-fermented HG and SG extracts, and fermentation had a greater effect on the antioxidant activity of HG than that of SG, because there was relatively small difference in antioxidant activities of SG, between before and after fermentation, except in DPPH radical scavenging activity. 

### 3.4. Cell Viability and Inhibitory Effect on NO Production

**Figure 3:**
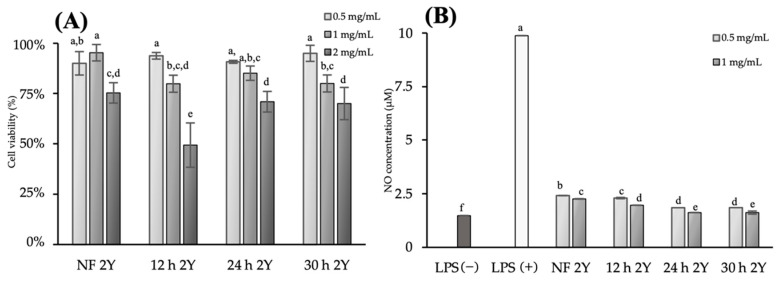
Cytotoxicity and inhibitory effect on nitric oxide production properties of 2-year-old hydroponic ginseng (5 mg/mL) during fermentation. (**A**) Cytotoxicity of RAW 264.7 cells under the treatment of hydroponic ginseng extract, (**B**) inhibition on nitric oxide production under the treatment of hydroponic ginseng extract. LPS, lipopolysaccharide; NF 2Y, non-fermented 2-year-old hydroponic ginseng; 12 h 2Y, 12 h fermented 2-year-old hydroponic ginseng; 24 h 2Y, 24 h fermented 2-year-old hydroponic ginseng; 30 h 2Y, 30 h fermented 2-year-old hydroponic ginseng. Values are represented as the mean ± standard deviation of triplicate experiments. The different letters on the error bars indicate statistically significant differences between sample groups (*p* < 0.05).

The effects of HG extracts on cell viability were evaluated using the MTT assay to determine the appropriate concentration of the ginseng extracts for the subsequent experiments and inhibitory effect on nitric oxide (NO) production. In Figure 3A, the viability of RAW 264.7 cells was above 80% in all groups under the concentration of 1 mg/mL, indicating that all concentrations from 0.5 to 1 mg/mL of HG extracts were not toxic to RAW 264.7 cells. However, 2 mg/mL HG extracts were toxic to RAW 264.7 cells. Therefore, 0.5 and 1 mg/mL HG extracts were used to evaluate its inhibitory effect on NO production. 

In Figure 3B, the positive control (LPS, 4 µg/mL) showed high NO production (9.88 ± 0.75 μM). Contrarily, fermented HG extracts significantly inhibited NO production. At a concentration of 1 mg/mL, non-fermented, 12 h fermented, 24 h fermented and 30 h fermented HG extracts inhibited NO production (2.26 ± 0.125 μM, 1.96 ± 0.12 μM, 1.62 ± 0.26 μM and 1.62 ± 0.03 μM, respectively). 24 h fermented HG extracts showed the highest Inhibitory effect on NO production of every fermented samples.

## 4. Discussion

In general, the roots of soil-cultivated ginseng have been used commercially as a traditional medicine in northeast Asia. Many researchers have reported that the root of 6-year-soil-cultivated ginseng has immune-reinforcing effects, anti-inflammatory effects and antioxidant activity [35,36,37]. These health functions of ginseng are related to various ginsenosides, including Rd, F2 and compound K, and phenolic compounds [38,39]. However, the composition of ginsenosides, phenols and flavonoids also varies depending on the type of ginseng or the ginseng cultivation method [40]. Hydroponic ginseng is known for its excellent health and functional properties because the composition of saponin, an active compound, is higher in hydroponic ginseng than soil-cultivated ginseng roots. In particular, phenol and flavonoid contents in HG extracts were approximately 19% and 18% higher than those in SG extracts, respectively. The ABTS assay revealed that HG extracts had 15% stronger activity than SG extracts. Indeed, the practical application of hydroponic ginseng has recently attracted attention in the pharmaceutical industry [26].

In this study, HG and SG were fermented for 24 h using *L. lactis* KC24 screened from kimchi. During fermentation, the ginseng extracts were not supplemented with other substrates to induce *L. lactis* KC24 to use sugars preferentially in ginseng extracts. To decide the time of fermentation, 6 h, 12 h, 18 h, 24 h and 30 h fermented samples were checked its bio-functionality. However, the antioxidant activity and inhibitory effect of NO production were decreased after 24 h fermentation. Therefore, 12 h fermented ginseng extract, which is in the middle of fermentation, and 24 h fermented ginseng extract were selected as our fermented samples.

Here, the SG extract, used as a reference group, also showed antioxidant activity, but it was lower than that of the fermented group. Fermentation increased total phenol and flavonoid contents in the extracts and the antioxidant activity. In particular, the HG extract fermented with *L. lactis* KC24 for 24 h showed the highest antioxidant activity among the extracts.

As summarized in Table 3, both the DPPH and ABTS radical scavenging assays showed increasing antioxidant activity after fermentation similarly, but the ABTS radical scavenging assay showed a significantly greater antioxidant activity than the DPPH radical scavenging assay. This pattern may be caused by the inherent properties of affinity for water, that is, ABTS and DPPH assays are hydrophilic and hydrophobic, respectively [41,42]. A hydrogen radical or electron is accepted by the ABTS cation to form a stable diamagnetic molecule and reduce free radicals. The results of the three antioxidant experiments were different because the principles of each experimental method were different. The increase in the antioxidant capacity of fermented HG extracts may be due to the increase in flavonoid and phenol contents as determined in previous studies, and not the bioconversion of ginsenosides [42,43]. Furthermore, the pattern of the inhibitory effect on NO production of the extracts was similar to that of the antioxidant activity, total phenol content and flavonoid content. The 24 h fermented HG extracts showed the highest functionality followed by the 12 h fermented HG extracts and did not show difference with 30 h fermented HG extracts.

These results may be because fermentation increased phenol and flavonoid contents in the ginseng extracts, improving the antioxidant ability and consequently inhibiting the production of NO, which causes inflammation in the body. As already mentioned, oxidative stress occurs when the level of ROS exceeds the antioxidant power of cells, and NO, a compound in which nitrogen is oxidized, is not processed, resulting in excessive secretion and cell apoptosis. Therefore, it is crucial to remove NO produced in the body and ameliorate the antioxidant ability to prevent the oxidation of cells. Moreover, if ginseng has such a function, it can also have anti-inflammatory effects because it is closely related to the anti-inflammatory reaction and immunity enhancement.

Collectively, the inhibitory effect of fermented hydroponic ginseng extract on NO production may be closely related to antioxidant activity and the content of flavonoids and phenolic compounds. In addition, the toxicity of hydroponic ginseng decreased as the fermentation time of the sample increased.

## 5. Conclusions

This study aimed to evaluate the antioxidant activity and inhibitory effects on NO production of all parts of HG fermented with *L. lactis* KC24, especially for 24 h. The experimental results suggested that fermented HG had greater antioxidant capacity and exhibited an inhibitory effect on NO production. These functionalities suggest new possibilities for the application of HG as a raw material for food and health functional foods in the future. However, the bioconversion of ginsenosides in HG during fermentation did not occur. Therefore, in-depth research is warranted to identify the component changes in HG that enhanced the antioxidant activity and inhibitory effect on NO production in addition to phenol and flavonoid contents during fermentation. Furthermore, hydroponic ginseng fermented with probiotics is expected to exhibit anti-inflammatory and immune enhancing activities. In addition, sensory evaluation studies that reflect consumer preferences in food applications are also expected to contribute to the industrialization of hydroponic ginseng.

## Figures and Tables

**Table 1:** Characteristics of *Lactococcus lactis* KC24 as a probiotics [20,21].

	Characteristics of Lactic Acid Bacteria
* Lactococcus lactis * KC24	Tolerance to artificial gastric juice and bile acid
	Production of enzyme (did not produce carcinogenic enzyme)
	Inhibition against *Listeria monocytogenes* and *Staphylococcus aureus*
	Anti-inflammatory effects through NO production
	Antioxidant effects (through FRAP assay and β-carotene bleaching assay)
	Neuroprotective effect

**Table 2:** Total phenol and flavonoid contents in 2-year-old hydroponic ginseng and 6-year-old soil-cultivated ginseng after fermentation (2.5 mg/mL).

Sample		Total Phenol and Flavonoid Content		
		Non-Fermentation	12 h	24 h
Total phenol content (mg of GAE ^1^/100 g)	2Y HG	25.06 ± 1.08 ^b^	31.17 ± 1.08 ^a^	29.14 ± 0.36 ^a^
	6Y SG	13.88 ± 0.36 ^c^	25.32 ± 2.16 ^b^	29.65 ± 1.08 ^a^
Total flavonoid content (mg of QE ^2^/100 g)	2Y HG	12.92 ± 1.01 ^b^	16.04 ± 1.01 ^a^	10.06 ± 0.73 ^c^
	6Y SG	2.28 ± 0.73 ^e^	5.13 ± 0.37 ^d^	5.65 ± 0.00 ^d^

^1^ GAE = gallic acid equivalents and ^2^ QE = quercetin equivalents. ^a–e^ Different superscripts in the identical parameters signify significant differences (*p* < 0.05). All values are mean ± standard deviation of triplicate analysis and analyzed using ANOVA. 2Y HG, 2-year-old hydroponic ginseng; 6Y SG, 6-year-old soil-cultivated ginseng.

**Table 3:** Antioxidant activity analysis of 2-year-old hydroponic ginseng and 6-year-old soil-cultivated ginseng after fermentation by antioxidant assays.

Antioxidant Assay	Sample		Fermentation Time		
			Non-Fermentation	12 h	24 h
DPPH radical scavenging activity (%)	5 mg/mL	2Y HG	32.57 ± 0.03 ^b^	36.92 ± 0.06 ^b^	41.06 ± 0.01 ^a^
		6Y SG	22.54 ± 0.03 ^d^	21.18 ± 0.02 ^d^	26.55 ± 0.02 ^c^
ABTS radical scavenging activity (%)	2 mg/mL	2Y HG	62.62 ± 0.00 ^d^	82.86 ± 0.01 ^a^	86.12 ± 0.06 ^b^
		6Y SG	54.00 ± 0.02 ^e^	59.96 ± 0.01 ^d^	72.46 ± 0.03 ^c^
Reducing power assay (L-Cysteine, µM)	5 mg/mL	2Y HG	14.94 ± 0.00 ^c^	15.17 ± 0.00 ^b^	15.36 ± 0.00 ^a^
		6Y SG	14.76 ± 0.00 ^d^	14.79 ± 0.00 ^d^	15.11 ± 0.00 ^b^
FRAP assay (µM FeSO_4_ equivalents)	5 mg/mL	2Y HG	35.41 ± 0.00 ^c^	36.12 ± 0.00 ^a^	35.86 ± 0.00 ^b^
		6Y SG	35.17 ± 0.00 ^d^	35.19 ± 0.00 ^d^	35.34 ± 0.00 ^c^

^a–^^e^ Different superscript letters in the same antioxidant assay signify significant differences (*p* < 0.05). Data are presented as mean ± standard deviation of triplicate experiments. 2Y HG, 2-year-old hydroponic ginseng; 6Y SG, 6-year-old soil-cultivated ginseng.

**Table 4:** IC_50_ of 2-year-old hydroponic ginseng and 6-year-old soil-cultivated ginseng after fermentation by DPPH and ABTS assays.

Antioxidant Assay	Sample	IC_50_		
		Non-Fermentation	12 h	24 h
DPPH radical scavenging activity	2Y HG	7.7 ± 0.03 ^c^	6.8 ± 0.06 ^d^	6.1 ± 0.01 ^d^
	6Y SG	11.1 ± 0.03 ^a^	11.8 ± 0.02 ^a^	9.4 ± 0.02 ^b^
ABTS radical scavenging activity	2Y HG	1.6 ± 0.00 ^a^	1.2 ± 0.01 ^c^	1.2 ± 0.06 ^c^
	6Y SG	1.9 ± 0.02 ^a^	1.7 ± 0.01 ^a^	1.4 ± 0.03 ^b^

^a–^^d^ Different superscript letters in the same antioxidant assay signify significant differences (*p* < 0.05). Data are presented as mean ± standard deviation of triplicate experiments. 2Y HG, 2-year-old hydroponic ginseng; 6Y SG, 6-year-old soil-cultivated ginseng.

## Data Availability

Data is contained within the article.
